# Level of education and multiple sclerosis risk over a 50-year period: Registry-based sibling study

**DOI:** 10.1177/1352458516646863

**Published:** 2016-07-11

**Authors:** Kjetil Bjørnevik, Trond Riise, Espen Benjaminsen, Elisabeth G Celius, Ole P Dahl, Margitta T Kampman, Kristin I Løken-Amsrud, Rune Midgard, Kjell-Morten Myhr, Øivind Torkildsen, Anita Vatne, Nina Grytten

**Affiliations:** Department of Global Public Health and Primary Care, University of Bergen, Bergen, Norway/The Norwegian Multiple Sclerosis Competence Center, Department of Neurology, Haukeland University Hospital, Bergen, Norway; Department of Global Public Health and Primary Care, University of Bergen, Bergen, Norway/The Norwegian Multiple Sclerosis Competence Center, Department of Neurology, Haukeland University Hospital, Bergen, Norway; Department of Neurology, Nordland Hospital Trust, Bodø, Norway; Department of Neurology, Oslo University Hospital, Ullevål, Oslo, Norway/Institute of Health and Society, Faculty of Medicine, University of Oslo, Oslo, Norway; Department of Neurology, Namsos Hospital, Namsos, Norway; Department of Neurology, University Hospital of North Norway, Tromsø, Norway; Innlandet Hospital Trust, Lillehammer, Norway; Molde Hospital, Molde, Norway/Unit for Applied Clinical Research, Norwegian University of Science and Technology, Trondheim, Norway; The Kristian Gerhard Jebsen Centre for MS Research, Department of Clinical Medicine, University of Bergen, Bergen, Norway/The Norwegian Multiple Sclerosis Registry and Biobank, Department of Neurology, Haukeland University Hospital, Bergen, Norway; The Norwegian Multiple Sclerosis Competence Center, Department of Neurology, Haukeland University Hospital, Bergen, Norway/The Kristian Gerhard Jebsen Centre for MS Research, Department of Clinical Medicine, University of Bergen, Bergen, Norway; Department of Neurology, Sørlandet Hospital Kristiansand, Kristiansand, Norway; The Norwegian Multiple Sclerosis Competence Center, Department of Neurology, Haukeland University Hospital, Bergen, Norway/The Kristian Gerhard Jebsen Centre for MS Research, Department of Clinical Medicine, University of Bergen, Bergen, Norway

**Keywords:** Multiple sclerosis, epidemiology, risk factors, socioeconomic status, education, environmental risk factors

## Abstract

**Background:**

The conflicting results from studies on socioeconomic status (SES) and multiple sclerosis (MS) risk might be due to a change in the distribution of environmental exposures over time or to methodological limitations in previous research.

**Objective:**

To examine the association between SES and MS risk during 50 years.

**Methods:**

We included patients registered in Norwegian MS registries and prevalence studies born between 1930 and 1979, and identified their siblings and parents using the Norwegian Population Registry. Information on education was retrieved from the National Education Registry, categorized into four levels (primary, secondary, undergraduate and graduate) and compared in patients and siblings using conditional logistic regression.

**Results:**

A total of 4494 MS patients and 9193 of their siblings were included in the analyses. Level of education was inversely associated with MS risk (*p* trend < 0.001) with an odds ratio (OR) of 0.73 (95% confidence interval (CI): 0.59–0.90) when comparing the highest and lowest levels. The effect estimates did not vary markedly between participants born before or after the median year of birth (1958), but we observed a significant effect modification by parental education (*p* = 0.047).

**Conclusion:**

Level of education was inversely associated with MS risk, and the estimates were similar in the earliest and latest birth cohorts.

## Introduction

Multiple sclerosis (MS) is a demyelinating disease of the central nervous system with unknown aetiology, and is one of few diseases, including some allergic and autoimmune diseases,^1^ that have been associated with higher socioeconomic status (SES). For MS, early studies observed a higher disease risk among professional workers than in unskilled workers, which suggested that exposures associated with higher SES increased disease risk.^2,3^ Similarly, Kurtzke and Page^4^ observed a positive association between SES and MS risk in a large nested case–control study. More recent studies, however, have not been able to reproduce these findings, and some have arrived at opposite conclusions.^5–7^ This could reflect a change over time in the distribution of exposures relevant for MS or country-specific differences in access to education and associations between education and such exposures. Still, the heterogeneity observed could also be due to methodological limitations in previous research. Several studies have used study designs that are prone to selection and recall bias, which may have contributed to the apparently conflicting results.

To address this, we conducted a large population-based study using national registries to collect objective and reliable information on level of completed education, a valid marker of SES,^8^ on close to 4500 MS patients, their siblings and their parents over a period of 50 years.

## Methods

### Study population and design

We used The Norwegian MS Registry^9^ as the primary source to identify MS patients. This registry was established in 2001, enrols MS patients in all of Norway and currently includes close to 6000 patients, which corresponds to approximately 60% of all MS patients in Norway. We further used the Oslo MS Registry,^10^ a registry that was established in 1990, which enrols MS patients living in Oslo and included approximately 1200 MS patients who were not registered in the national MS registry at the time of data extraction. Moreover, available prevalence and hospital record studies^11–15^ were used to identify patients not registered in the registries, as described in previous studies using the same cohort of patients.^16,17^ All patients had been diagnosed according to the criteria of Poser et al.^18^ or McDonald^19^ and were born between 1930 and 1979. In total, the cohort included 6928 MS patients.

Identified patients were linked to the Norwegian Population registry (Statistics Norway). This registry was established in 1964 and includes national identification numbers for the total Norwegian population alive since 1960.^20^ We used the patients’ identification number to retrieve information on sex and birth year of their unaffected siblings and parents. A total of 9346 siblings without MS, 5711 mothers and 5611 fathers were identified and included in the cohort. The number of siblings per MS patients ranged from 1 to 13. The siblings were included as controls, while the parents were included in analyses of effect modification. The national identification numbers of all study participants were then linked to the National Education Registry, and information on levels of completed education was retrieved for all participants for whom this information was available. This registry was established in 1970 and contains information on the highest level of completed education on the total Norwegian population alive on or after this year.^21^ Use of the national identification number ensured that no participant was included more than once.

Due to the dependency between the cases and controls in our study, the educational level of each MS case was compared to the level of their own siblings. Thus, patients with no siblings were excluded from the analyses. A total of 4502 MS patients had one or more siblings. Among those, information on completed education was available for 4494 patients. Educational level was available for 9193 of the siblings. In the families that were included in the analyses, educational level was available for 4297 of the fathers and 4384 of the mothers. Parental education from at least one of the parents was available for 4429 of these families.

### Statistical analysis

Categories for highest level of completed education were generated based on the information provided by the National Education Registry. The new variable took value 1 for primary level (10 years or less), 2 for secondary level (11–13 years), 3 for undergraduate level (14–17 years) and 4 for graduate level (18 years or more). A variable that combined parental education was generated, and it took level 1 if at least one of the parents had higher education and 0 otherwise. Higher education was defined as undergraduate and graduate levels.

The association between disease and exposure was estimated as odds ratios (OR) with 95% confidence intervals (CIs) matching the cases with their siblings using conditional logistic regression. Level of education was included as a categorical variable in this model, and we compared the different levels of education to the reference level, which was the lowest level. As we are using a conditional regression model comparing cases with their own siblings, the analyses are adjusted for parental education and family size by design. In a multivariate model, we adjusted for birth order (1, 2, 3 and >3) and residency at birth (North and South). North was defined as one of the three most Northern counties in Norway (Nordland, Troms and Finmark) and South was defined as any of the other counties. We tested for interaction on the multiplicative scale by introducing an interaction term into the regression model. Specifically, we tested for interaction between level of education and sex, parental education and time periods for year of births. The time periods included birth years before and after 1958, which was the median birth year for the participants. We tested for *p* trend by including level of education as a continuous variable in the model. All analyses were adjusted for sex and age in 5-year intervals.

The statistical analyses were performed in Stata Statistical Software: Release 14. College Station, TX: StataCorp 2015.

### Ethical approval

The study was approved by the Regional Committee for Medical and Health Research Ethics (REK Nord).

## Results

Table 1 provides the baseline characteristics of the study population according to the level of education. Those with highest level of education had fewer siblings and were more likely to be first born and to have parents with higher education. The overall distribution of level of education among patients, siblings and parents is provided in Table 2.

**Table 1.** Baseline characteristics of the study population according to the level of education in a large registry-based sibling study in Norway.

	Primary	Secondary	Undergraduate	Graduate
Cases				
No. of participants	1003	2186	1050	255
Year of birth, mean	1958.3	1959.2	1961.8	1961.8
No. of siblings, mean	2.2	2.0	2.0	1.9
First born, %	31.4	37.9	39.5	41.2
Female, %	64.7	63.7	75.2	58.0
Born in North tier,^a^ %	14.3	12.6	14.2	11.7
Parents with higher education,^b^ %	4.6	11.0	29.0	50.8
Controls				
No. of participants	2104	4431	2063	595
Year of birth, mean	1958.6	1959.5	1961.5	1961.3
No. of siblings, mean	3.0	2.8	2.6	2.5
First born, %	27.7	31.3	32.7	35.0
Female, %	47.6	44.7	59.6	36.0
Born in North tier,^a^ %	15.0	14.3	17.6	12.5
Parents with higher education,^b^ %	5.0	9.7	26.4	46.6

a Born in one of the three most Northern counties in Norway (Nordland, Troms and Finmark).

b Defined as at least one parent with undergraduate or graduate level of education.

**Table 2.** Overall distribution of educational level among MS patients, their siblings and their parents in a large registry-based sibling study in Norway.

	Level of education				
	Primary	Secondary	Undergraduate	Graduate	Missing^a^
Cases, %	22.3	48.6	23.3	5.7	0.2
Family					
Siblings, %	22.5	47.4	22.1	6.4	1.6
Mothers, %	49.2	40.6	7.2	0.4	2.6
Fathers, %	40.5	42.5	8.0	4.4	4.6

MS: multiple sclerosis.

a Missing values on level of education.

We found a statistically significant inverse association between level of education and risk of MS (*p* trend < 0.001; Table 3). Participants with the highest level of education had a lower MS risk compared to those with the lowest level (OR: 0.73, 95% CI: 0.59–0.90). Undergraduate level of education was also significantly associated with lower MS risk (OR: 0.82, 95% CI: 0.72–0.95).

**Table 3.** The OR of MS according to level of education in a large registry-based sibling study in Norway.

	No. of cases/controls	OR (95% CI)^a^	
		Multivariate adjusted^b^	Multivariate adjusted^c^
Level of education			
Primary	1003/2104	Ref.	Ref.
Secondary	2186/4431	0.96 (0.87–1.07)	0.96 (0.86–1.07)
Undergraduate	1050/2063	0.82 (0.72–0.95)	0.82 (0.72–0.95)
Graduate	255/595	0.73 (0.59–0.90)	0.73 (0.59–0.90)
*p* trend		<0.001	<0.001

MS: multiple sclerosis; OR: odds ratio; CI: confidence interval.

a Effect estimates calculated using conditional logistic regression comparing level of education in patients with the level of their own siblings.

b Adjusted for age, sex, parental education and number of siblings.

c Further adjusted for birth order and residency at birth.

We observed significant effect modification by parental education (*p* = 0.047). The highest level of education was significantly associated with a decreased MS risk among participants who had no parents with higher education (OR highest vs lowest level: 0.65, 95% CI: 0.50–0.84; Table 4), but not among those with one or more parents with higher education (OR highest vs lowest level: 1.29, 95% CI: 0.79–2.09; Table 4). We further restricted the analyses to those born before and after the median year of birth (1958), and observed that there was only a significant effect modification by parental education in the last period (*p* = 0.006). There was no significant difference between women (OR highest vs lowest level: 0.61, 95% CI: 0.43–0.85) and men (OR highest vs lowest level: 0.81, 95% CI: 0.54–1.21) (*p* for effect modification = 0.36). Similarly, there was no significant difference between those born before 1958 (OR highest vs lowest level: 0.72, 95% CI: 0.50–1.03; Table 5) and after 1958 (OR highest vs lowest level: 0.84, 95% CI: 0.63–1.12), (*p* for effect modification = 0.49).

**Table 4.** The OR of MS according to level of self-education and parental education^a^ in a large registry-based sibling study in Norway.

	Low parental education		High parental education	
	No. of cases/controls	OR (95% CI)^b^	No. of cases/controls	OR (95% CI)^b^
Level of education				
Primary	924/1959	Ref.	44/103	Ref.
Secondary	1926/3968	0.94 (0.84–1.06)	237/424	1.50 (0.98–2.31)
Undergraduate	741/1513	0.80 (0.69–0.93)	303/542	1.28 (0.82–1.98)
Graduate	125/316	0.65 (0.50–0.84)	129/276	1.29 (0.79–2.09)
*p* trend		<0.001		0.94

MS: multiple sclerosis; OR: odds ratio; CI: confidence interval.

a Defined as at least one parent with undergraduate or graduate level of education.

b Effect estimates calculated using conditional logistic regression comparing level of education in patients with the level of their own siblings. All estimates are adjusted for age, sex and number of siblings.

**Table 5.** The OR of MS according to time period of birth in a large registry-based sibling study in Norway.

	Born in or before 1958		Born after 1958	
	No. of cases/controls	OR (95% CI)^a^	No. of cases/controls	OR (95% CI)^a^
Level of education				
Primary	459/976	Ref.	544/1128	Ref.
Secondary	1082/2140	1.00 (0.84–1.20)	1104/2291	1.00 (0.85–1.17)
Undergraduate	386/825	0.81 (0.64–1.04)	664/1238	0.87 (0.72–1.07)
Graduate	89/240	0.72 (0.50–1.03)	166/355	0.84 (0.63–1.12)
*p* trend		0.04		0.11

MS: multiple sclerosis; OR: odds ratio; CI: confidence interval.

a Effect estimates calculated using conditional logistic regression comparing level of education in patients with the level of their own siblings. All estimates are adjusted for age, sex, parental education and number of siblings.

## Discussion

We observed that the level of completed education was inversely associated with MS risk in Norway. The association did not vary significantly during the study period of 50 years.

Our finding of an inverse relationship between level of education and MS risk is consistent with several recent studies. A large Norwegian prospective study with close to 400,000 participants observed a higher MS risk among the participants with lowest level of education.^5^ These findings were later replicated by two case–control studies from the United States and Norway.^6,7^

Our observations suggest that currently unknown exposures are driving the association between education and MS. The prevalence of smoking has declined substantially over the study period, and the distribution of the habit across levels of SES has changed. In the 1950s, the majority of Norwegian doctors were smokers,^22^ while smoking is primarily a habit associated with lower SES today. Thus, if smoking were driving the association, we would expect to observe differences between patients born before and after 1958. Furthermore, late primary infection of Epstein–Barr virus (EBV), which increases the risk of infectious mononucleosis (IM),^23^ is associated with higher SES^24^ and can therefore not explain our observations. Two recent studies observed an inverse association between education and MS that persisted after adjustment for currently known risk factors,^6,7^ which is consistent with our findings.

Sibling studies are prone to overmatching as siblings share a substantial amount of both genetic and environmental exposures, which reduce the power to detect differences between cases and controls. This adds weight to our findings, as we still observe a significant association between education and MS. Since siblings are likely to share fewer exposures the older they get, our findings could suggest that education is a marker of an exposure in or after the adolescence. This is consistent with studies on other known risk factors, including vitamin D^25^ and EBV,^26^ which have observed that exposure during adolescence may be especially important for future MS risk. It has recently been suggested that sodium intake^27^ and the intestinal microbiome^28^ could be relevant for later MS risk. Although these suggestions are mainly based on animal studies, both these factors could be associated with SES and provide biologically plausible pathways for a subsequently altered MS risk. Furthermore, low SES itself has also been associated with increased proinflammatory signalling, including higher levels of the proinflammatory cytokine interleukin 6 (IL-6),^29^ which is likely to be important for the development of autoimmune diseases.^30^

We observed a significant effect modification by parental education. While there was an inverse association between education and MS risk among those with parents with lower levels of education, there was no significant association in families with higher parental education. This could reflect early life exposures associated with higher SES that compete with other risk factors later in life that are associated with lower SES. A large Danish cohort study reported a lower MS risk among children of mothers with higher education, but this study investigated the association between cases and unrelated controls.^31^ Moreover, a case–control study in Northern California reported that both low parental and low self-education were associated with a higher MS risk.^6^ Overall, this could suggest that both childhood and adulthood SES are important for subsequent MS risk. Our study was not powered to test for effect modification of the education level of mothers and fathers separately.

The use of national registries for exposure information is a strength in this study, as we are not relying on recalled or self-reported information. Furthermore, as the registries contain information on the entire Norwegian population, we have complete information on all siblings of the patients. This is important for the validity of the findings, as studies on education and disease are especially prone to bias when there is a low participation rate among the participants. The likelihood of agreeing to take part in a study is affected by the educational level of those invited,^32^ which could induce a selection of highly educated participants into the study, especially in the less-motivated control group in a case–control study design. Furthermore, another strength of this study is that access to education was gradually equally distributed over the study period by the government agency, The Norwegian State Educational Loan Fund, thus providing universally covered and equal financial access to education.

Our study has some limitations. First, we had no information on relevant environmental exposures, like smoking, and are thus not able to adjust for this in the analysis. Still, our findings are consistent with two recent studies that were able to adjust for these.^6,7^ Second, as we did not have access to year of MS onset, our results could be affected by reverse causality. If a patient has an early onset of disease, it could affect their ability take part in higher education, as cognitive impairment is common early in the course of the disease.^33^ However, early onset of disease did not seem to drive the association between education and MS in previous studies.^6,7^ Furthermore, we observed a similar association with undergraduate level of education, which makes it unlikely that reverse causality can fully explain our findings. Lastly, we cannot exclude the possibility that some participants died before the educational registry was established, which would lead to a misclassification of their educational level. Still, we observed similar results in both time periods examined, which makes it unlikely that this potential misclassification play a major role in the results.

In conclusion, we observed an inverse association between level of completed education and MS risk that persisted over the whole study period of 50 years in Norway. This suggests that there has not been a shift in the direction of the association in Norway during this period.
